# Application of the Orthogonal Polynomial Fitting Method in Estimating PM_2.5_ Concentrations in Central and Southern Regions of China

**DOI:** 10.3390/ijerph16081418

**Published:** 2019-04-19

**Authors:** Bingtian Li, Yongzhi Liu, Xinyi Wang, Qingjun Fu, Xianqing Lv

**Affiliations:** 1 College of Ocean Science and Engineering, Shandong University of Science and Technology, Qingdao 266590, China; skd994650@sdust.edu.cn (B.L.); fuqingjun@sdust.edu.cn (Q.F.); 2 Physical Oceanography Laboratory/CIMST, Ocean University of China and Qingdao National Laboratory for Marine Science and Technology, Qingdao 266200, China; 3 First Institute of Oceanography, Ministry of Natural Resources and Laboratory for Regional Oceanography and Numerical Modeling, Qingdao National Laboratory for Marine Science and Technology, Qingdao 266061, China; yzliu@fio.org.cn (Y.L.); wangxy@fio.org.cn (X.W.)

**Keywords:** fine particles (PM_2.5_), orthogonal polynomial fitting (OPF), interpolation

## Abstract

Sufficient and accurate air pollutant data are essential to analyze and control air contamination problems. An orthogonal polynomial fitting (OPF) method using Chebyshev basis functions is introduced to produce spatial distributions of fine particle (PM_2.5_) concentrations in central and southern regions of China. Idealized twin experiments (IE1 and IE2) are designed to validate the feasibility of the OPF method. IE1 is designed in accordance with the most common distribution of PM_2.5_ concentrations in China, whereas IE2 represents a common distribution in spring and autumn. In both idealized experiments, prescribed distributions are successfully estimated by the OPF method with smaller errors than kriging or Cressman interpolations. In practical experiments, cross-validation is employed to assess the interpolation results. Distributions of PM_2.5_ concentrations are well improved when OPF is applied. This suggests that errors decrease when the fitting order increases and arrives at the minimum when both orders reach 6. Results calculated by the OPF method are more accurate than kriging and Cressman interpolations if appropriate fitting orders are selected in practical experiments.

## 1. Introduction

Fine particles (PM_2.5_) are particulate matters suspended in the air with diameters within 2.5 μm. PM_2.5_ is usually reported as a principal pollutant of air contamination. Exposure to highly concentrated PM_2.5_ affects the respiratory and cardiovascular systems and might cause heart and lung disease [1,2]. Moreover, PM_2.5_ is thought to be involved in some processes of neurodegeneration [3]. It was estimated that the global premature mortality rate associated with PM_2.5_ was at 3.15 million per year in 2010, with China being the leading country at about 1.33 million [4]. PM_2.5_ in 2015 contributed as much as 40.3% to total stroke deaths, 26.8% to ischemic heart disease deaths and 23.9% to lung cancer deaths [5]. The threat of PM_2.5_ pollution on public health has become increasingly remarkable. Therefore, there has been continued interest in computing and predicting the concentrations of PM_2.5_ and its effects on human health [6,7]. Spatial variation of pollutant concentration fields should be taken into account when computing air pollutant concentrations [8,9]. Estimations of accurate spatial variations in PM_2.5_ concentrations will provide valuable information for health risks and air quality control programs. Sufficient and accurate pollutant data are required to analyze and control air contamination problems. However, the observations of PM_2.5_ concentration are limited. Observational data can only be obtained from monitoring stations which are distributed sparsely in space [10,11]. In fact, comparatively accurate and sufficient PM_2.5_ data can be estimated with the help of interpolation for use in air pollution investigations and its effects on health.

Spatial interpolation techniques are essential for estimating PM_2.5_ variations. Many interpolation methods, such as kriging and Cressman interpolations, have been widely used in atmospheric subjects [8,9,12,13,14]. Lee et al. [12] developed a space-time geostatistical kriging model to predict PM_2.5_ fields over continental United States and found the kriging estimate was more accurate for locations near monitoring stations. Sampson et al. [13] introduced a regionalized national universal kriging model for estimating annual averaged PM_2.5_ concentrations across the U.S. Their universal kriging model provides the basis for prediction at arbitrary spatial locations. Physick et al. [8] estimated exposure to ambient concentrations based on Cressman interpolation. 

With increasing application of spatial interpolation methods, there is a growing concern about accuracy and precision [15,16,17]. Kriging and Cressman interpolations are two kinds of interpolation methods widely used in various fields because of their high accuracy—but there are still defects in these methods. The kriging interpolation provides a description of data spatial structure and variance estimation, which depends on the expression of spatial variation of the property in terms of the variogram [10]. However, it is time-consuming and cumbersome because of the need to calculate inverse-matrices [18]. Cressman interpolation, in which the spatial influence of an observation is weighted as a function of distance, is flexible and easy to implement [19]. When Cressman interpolation is applied to datasets with large distances between grid points, it produces relatively large errors [15,17]. So, Cressman interpolation needs to be modified to ensure that the interpolated result is close to the observation [20,21]. Some interpolation methods, such as spline interpolation, radial basis function interpolation and orthogonal polynomial fitting (OPF), are widely used in the fields of artificial intelligence, image processing and oceanic subjects [16,17,22,23]. Those methods are more accurate and efficient and need to be introduced to applications with atmospheric subjects. Orthogonal polynomial fitting is one of the methods combining accuracy with efficiency. The application of orthogonal polynomial fitting has been studied for decades [24,25,26,27,28,29,30]. In our study, an orthogonal polynomial fitting method based on Chebyshev basis functions is introduced to estimate PM_2.5_ concentrations in central and southern regions of China. The details of this method are shown in part of Appendix A. The paper is organized as follows. In Section 2 the monitoring data of PM_2.5_ concentrations are introduced. In Section 3, twin idealized experiments in central and southern China are performed. Practical experiments are also carried out in this section. Section 4 presents the conclusions.

## 2. Monitoring Data

**Figure 1:**
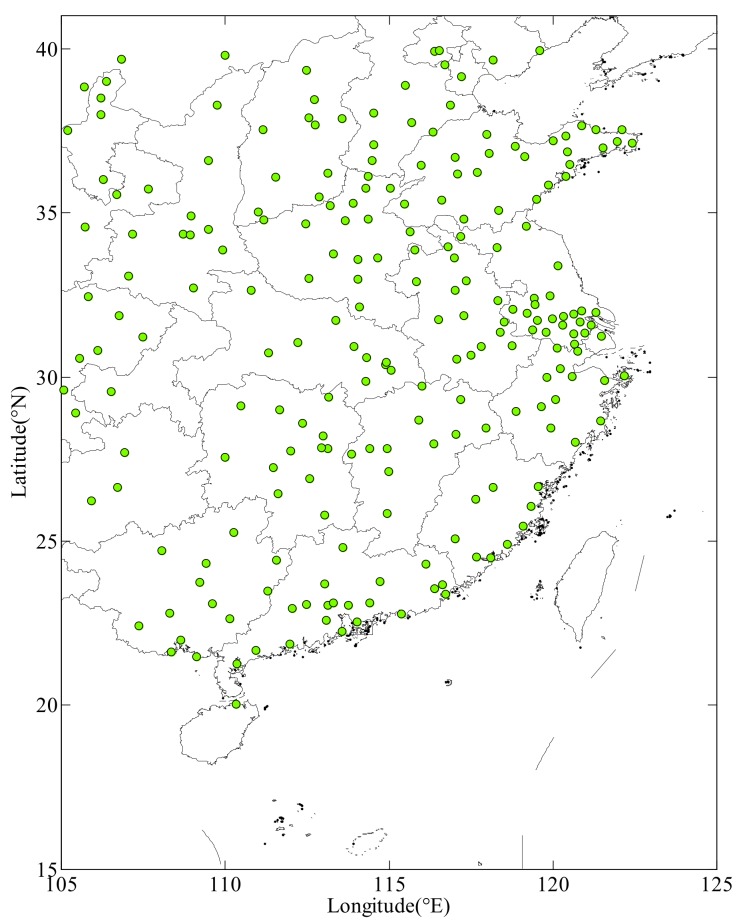
Distribution of monitoring cities.

The monitoring data are from the China National Environmental Monitoring Center. The purpose of observation is to investigate the temporal/spatial distribution and variation of PM_2.5_, PM_10_ and various air pollution factors in China, to assess and evaluate the current situation and the variation trend of the atmospheric environment and to diagnose the air pollution problems. Daily averaged urban air quality observation data are provided by the China National Environmental Monitoring Center. There are more than 1400 stations monitoring the air quality of urban areas. Monitoring stations are well distributed in the districts of 337 cities. The PM_2.5_ concentration of a city is obtained by averaging the data from monitoring stations within that city. The number of cities located in the study zone is 225. Data of those 225 cities are used in this study. The observation period is a year (from January to December 2017). Data from 27 December 2017, 17 November 2017 and 3 October 2017 are analyzed in practical experiments. The distribution of the monitored cities is shown in Figure 1.

## 3. Results and Discussion

### 3.1. Idealized Twin Experiments

**Figure 2:**
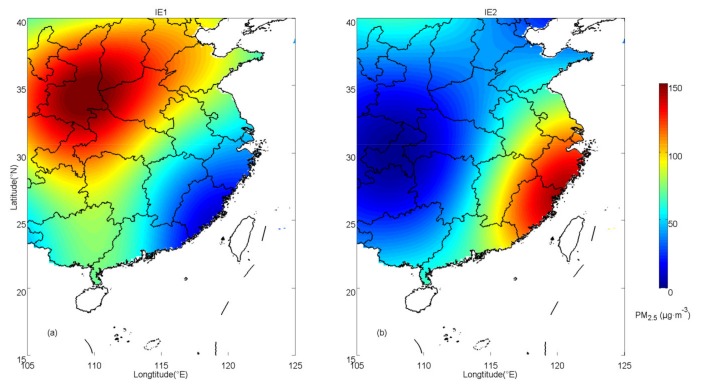
Prescribed distributions of PM_2.5_ concentrations for (**a**) idealized experiment 1 (IE1) and (**b**) idealized experiment 2 (IE2).

Two types of PM_2.5_ distributions are employed to evaluate the effectiveness of the OPF method. In idealized experiment 1 (IE1), PM_2.5_ concentrations are prescribed as one heavy-contamination center located in the northwest region and one slight-contamination center in the southeast region, which is the most common air pollution distribution in China according to observations. In idealized experiment 2 (IE2) the concentrations are prescribed according to a common distribution in spring and autumn. In IE2, the heavy-contamination center is located in the southeast region and the slight-contamination center is in the southwest region. The distributions of IE1 and IE2 are shown in Figure 2a,b, respectively.

For the Cressman interpolation, the influence radius is initially set to 4°. If there are at least five monitored cities within the influence radius of a grid, the PM_2.5_ concentration of that grid will be computed. Otherwise the influence radius is increased by 0.1° until there are enough cities within the influence area. For the kriging interpolation, the semivariogram model in our study is chosen as a spherical semivariogram model. The range and sill were set to 10° and 1°, respectively. For the OPF method, the results theoretically become more accurate with an increase in polynomial order, if there are enough observational data. In fact, the amount of data available for analysis is limited and therefore polynomial orders should be selected in a reasonable range according to observations. The range of orders in this paper is limited to 10. In the idealized twin experiments, the orders of *x* and *y* directions are set to 5. The relationship between the selection of polynomial orders and the accuracy of estimation is discussed in the practical experiments section.

**Figure 3:**
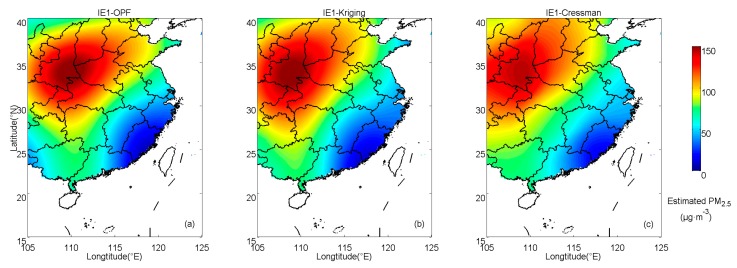
Interpolation results estimate by (**a**) orthogonal polynomial fitting (OPF), (**b**) kriging and (**c**) Cressman methods in IE1.

**Figure 4:**
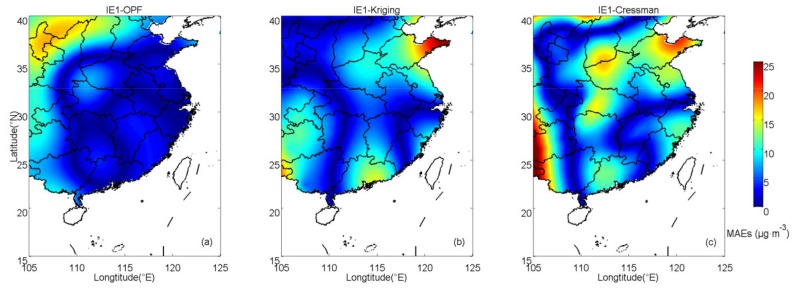
Spatial distributions of absolute errors of PM_2.5_ in IE1 with (**a**) OPF, (**b**) kriging and (**c**) Cressman methods.

In IE1, the interpolation results estimated by OPF, kriging and Cressman methods are shown in Figure 3. Spatial distributions of absolute errors of PM_2.5_ in IE1 are shown in Figure 4. For the OPF method, large deviations (>15 μg·m^−3^) only appear in the northwest region and there is no region with errors larger than 20 μg·m^−3^. For kriging interpolation, large deviations are mainly concentrated in Shandong peninsula, which is located in the northeast of the study zone. For Cressman interpolation, errors in Shandong peninsula as well as the southwest region of the study zone are relatively large. The mean absolute error (MAE) between estimated results and prescribed values at sampling points is often used as a precision indicator for interpolation methods [27]. The spatially averaged MAEs of OPF, kriging and Cressman interpolation results are 4.89, 5.91 and 7.87 μg·m^−3^, respectively. This suggests that the OPF method produces a better result than the kriging and Cressman interpolations, which estimates the PM_2.5_ concentrations in IE1 to be closest to the prescribed field.

**Figure 5:**
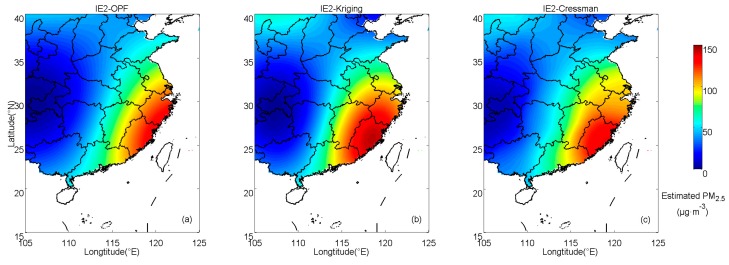
Interpolation results estimated by (**a**) OPF, (**b**) kriging and (**c**) Cressman methods in IE2.

**Figure 6:**
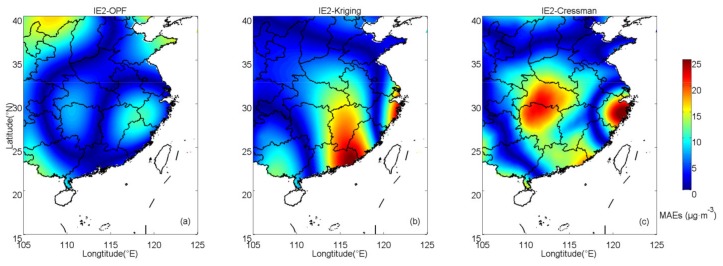
Spatial distributions of absolute errors of PM_2.5_ in IE2 with (**a**) OPF, (**b**) kriging and (**c**) Cressman methods.

**Figure 7:**
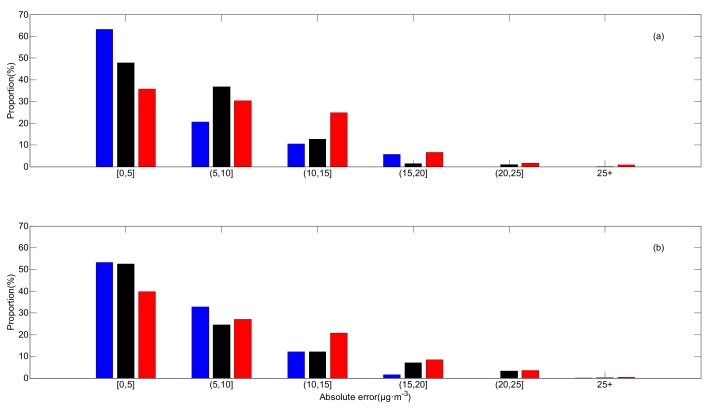
Components of absolute errors in (**a**) IE1 and (**b**) IE2. Blue: the OPF method, black: kriging interpolation and red: Cressman interpolation.

In IE2, the estimated results calculated by those methods are shown in Figure 5. Distributions of absolute errors are displayed in Figure 6. In IE2, the spatially averaged MAEs of OPF, kriging and Cressman estimations are 5.32, 6.56 and 7.84 μg·m^−3^, respectively. This suggests that the result estimated by the OPF method, in which the MAE is the smallest among those methods, is the closest to the prescribed field. Figure 7b shows components of absolute errors in IE2 and the corresponding results in IE1 are shown in Figure 7a. With the OPF method, more than 50% of the errors are smaller than 5 μg·m^−3^ in IE2 and small errors (≤5 μg·m^−3^) explain nearly 70% of the errors in IE1. The amount of large deviations with the OPF method is apparently less than those with kriging or Cressman interpolation in both IE1 and IE2.

The root mean square error (RMSE) between the estimated results and prescribed values is another indicator evaluating the accuracy of interpolation results [31]. The MAE represents the average distance between estimations and observations. Using MAE as an indicator can avoid canceling out errors with opposite signs, making it relatively accurate in reflecting the magnitude of errors. However, MAE is not sensitive to large errors. RMSE, which is more sensitive to large errors, is based on the sum of squared errors. Small amounts of very large errors will result in an obvious increase in the RMSE. The MAE tends to be smaller than the RMSE because the RMSE penalizes large errors while the MAE gives the same weight to all errors, whereas the sensitivity of the RMSE to outliers may induce ambiguities [32]. On the other hand, MAE is a natural measure of average error magnitude and that means it is an unambiguous measure. By employing both MAE and RMSE as indicators, the accuracy of the estimated results can be validated comprehensively. 

**Figure 8:**
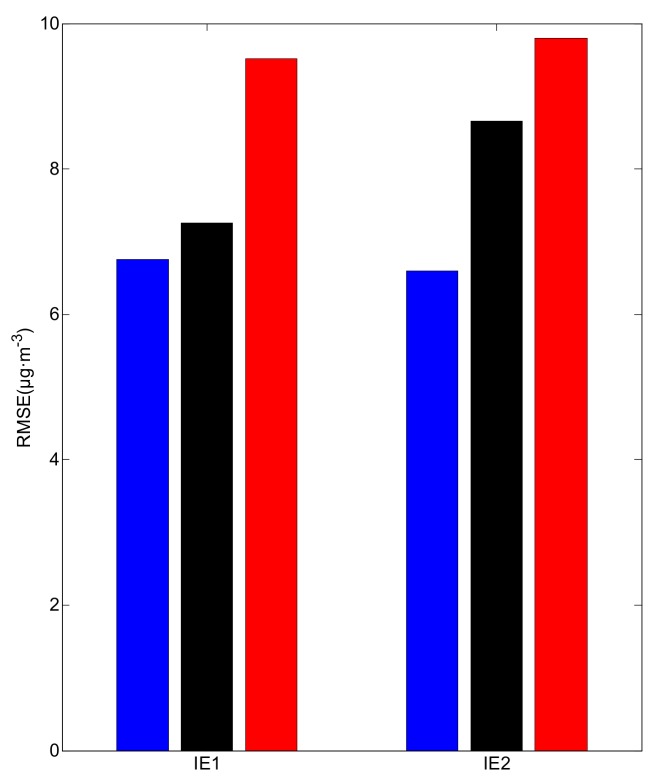
The root mean square error (RMSE) in idealized twin experiments. Blue: the OPF method, black: kriging interpolation and red: Cressman interpolation.

The RMSEs of the idealized twin experiments are shown in Figure 8. With the OPF method, the RMSE in IE1 is slightly larger than that of IE2, owing to the existence of larger errors (>15 μg·m^−3^ in MAE). With kriging or Cressman interpolation, the RMSEs in both idealized experiments are larger than those with the OPF method, indicating the accuracy of OPF, which agrees well with the result of the MAE analysis. The results of the idealized twin experiments reveal that the OPF method reconstructs PM_2.5_ distributions closer to the prescribed field than kriging or Cressman interpolation. This suggests that the estimation will apparently be more accurate if the OPF method is adopted.

### 3.2. Practical Experiments

As described in Section 3.1, OPF is an effective method to recover the prescribed PM_2.5_ concentrations, which encourages us to further apply this method to practical cases. Cross-validation is employed to assess the interpolation results [33]. In this study, the PM_2.5_ monitoring dataset is randomly split into eight subsets. In each subset, the data of 15 observed cities, which are well distributed in the study zone, are selected as set A, while the data of the other cities are referred to as set B. The data in set B are used to interpolate, meanwhile the accuracy of estimation is evaluated with the data in set A. Cross-validation is repeated eight times, represented by CV1 to CV8 in each practical experiment. 

**Figure 9:**
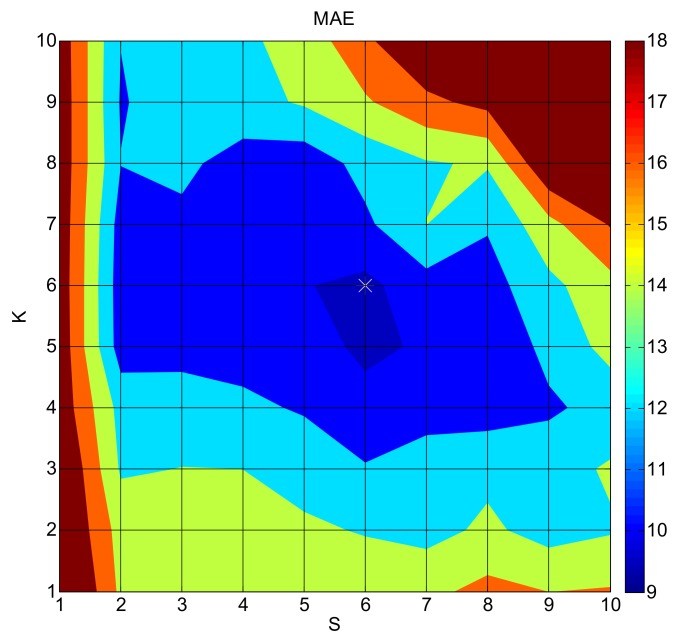
The mean absolute error (MAE) variation (μg·m^−3^) with orders in practical experiment 1 (PE1) (the marker represents the minimum MAE when s and k are both 6).

Practical experiment 1 (PE1) represents the experiment estimating distribution of PM_2.5_ concentrations on 2017 December 27. With the OPF method, PM_2.5_ distributions are calculated 100 times (polynomial orders of *x* and *y* directions each increase from 1 to 10) in the processes of cross-validation. Results and MAEs of the validation cities are averaged. The errors in PE1 are obtained by averaging the MAEs from all the cross-validations. Figure 9 demonstrates the MAE variation with orders. The MAEs decrease with the polynomial orders increasing and arrive at the minimum when s and k (polynomial orders of *x* and *y* directions) are both set to 6. The results suggest that errors are large when fitting orders are low and errors become extremely large when high polynomial orders are employed. Amplifying polynomial orders involves calculating much more numbers of polynomial coefficients, requiring a large amount of monitoring data. However, the amount of data is insufficient to support the accurate calculation of all the polynomial coefficients. The deviation of polynomial coefficients induces large errors in the results. It can be speculated that the estimations will become much closer to the observation when high-order OPF is applied, if enough monitoring data are available. In circumstances with limited numbers of observations, the polynomial orders should be selected within a reasonable range.

**Figure 10:**
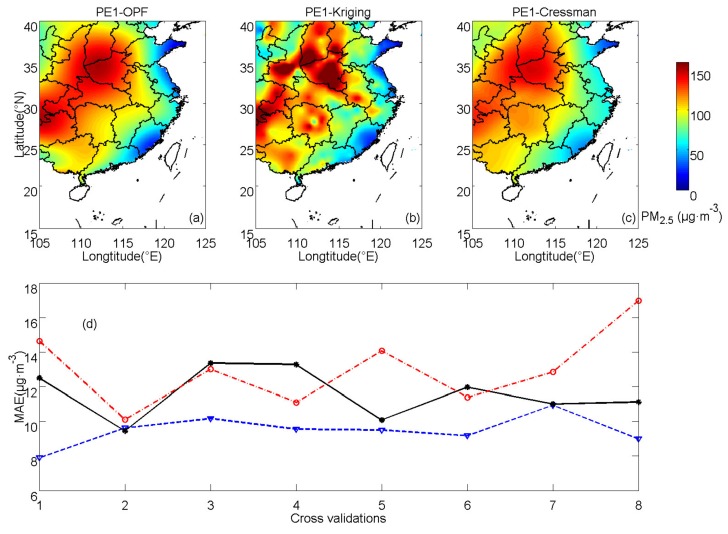
PM_2.5_ concentrations of PE1 calculated by (**a**) OPF, (**b**) kriging, (**c**) Cressman methods and (**d**) averaged MAEs for the corresponding methods (OPF method: blue dashed line, kriging interpolation: black solid line and Cressman interpolation: red dash-dot line).

PE1 is continued with fitting orders (s and k) both set to be 6 with the aim of producing relatively accurate results. The averaged MAE is 9.48 μg·m^−3^ (close to that in CV5) of the OPF method. Figure 10a demonstrates the OPF estimations of PM_2.5_ concentrations in CV5. The averaged MAE of kriging interpolation is 11.60 μg·m^−3^, which is close to that in CV6. For Cressman interpolation, the averaged MAE is 13.02 μg·m^−3^, close to that in CV4. Figure 10b,c represent interpolation results computed by kriging and Cressman interpolations, respectively. The estimation with the OPF method presents lower errors than those with kriging and Cressman interpolations in PE1. Averaged MAEs of all the cross-validations estimated by those three methods are shown in Figure 10d. The results estimated by the OPF method are more accurate than those estimated by kriging interpolation in most of the cross-validation processes except for CV2, in which the difference between those two methods is relatively small. The MAEs of Cressman interpolation are always larger than those of OPF in all the eight cross-validation processes. The results of those methods show similar spatial patterns, yet differences also exist. PM_2.5_ concentrations calculated by Cressman interpolation lose the capability of estimating detail of distributions. The kriging interpolation presents an unsmooth pattern with a larger MAE than the OPF method. 

**Figure 11:**
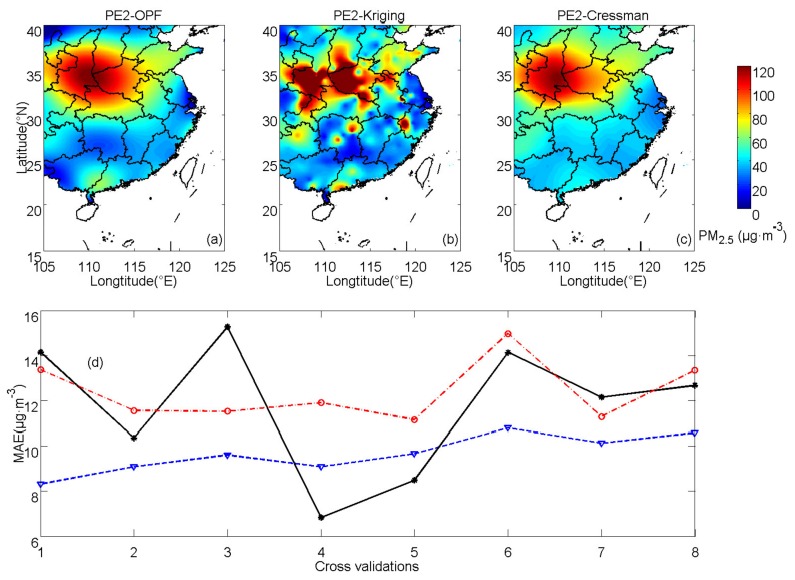
PM_2.5_ concentrations of practical experiment 2 (PE2) calculated by (**a**) OPF, (**b**) kriging and (**c**) Cressman methods and (**d**) averaged MAEs for the corresponding methods (OPF method: blue dashed line, kriging interpolation: black solid line and Cressman interpolation: red dash-dot line).

**Figure 12:**
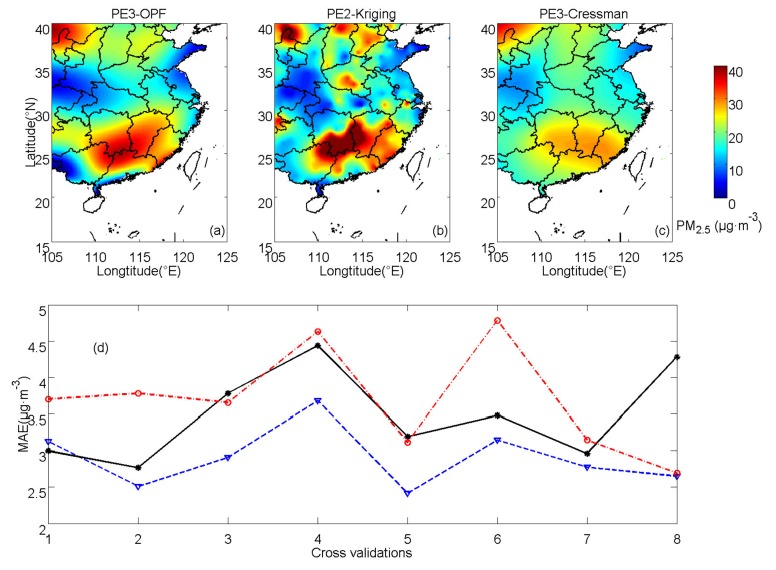
PM_2.5_ concentrations of practical experiment 3 (PE3) calculated by (**a**) OPF, (**b**) kriging and (**c**) Cressman methods and (**d**) averaged MAEs for the corresponding methods (OPF method: blue dashed line, kriging interpolation: black solid line and Cressman interpolation: red dash-dot line).

The OPF method is applied to practical experiments PE2 and PE3 (using the results of 17 November 2017 and 3 October 2017). PE2 represents cases with heavy pollution, whereas PE3 represents cases with slight pollution. In both PE2 and PE3, fitting orders are the same as those in PE1 (s = 6, k = 6). Figure 11 demonstrates the results of PE2 and estimations of PE3 are shown in Figure 12. In PE2, spatially averaged MAEs of OPF, kriging and Cressman interpolations are 9.66 11.76 and 12.39 μg·m^−3^, respectively. In PE3, the corresponding MAEs are 2.90, 3.49 and 3.69 μg·m^−3^, respectively. In PE2, the errors of the OPF method are lower than those of kriging interpolation in most of the cross-validation processes except for CV4 and CV5. The errors of Cressman interpolation are larger than those of the OPF method in all of the cross-validation processes. In PE3, the averaged MAEs of the OPF method are also smaller than those of kriging and Cressman interpolations in most of the cross-validation processes, which is consistent with results of PE1 and PE2. 

**Figure 13:**
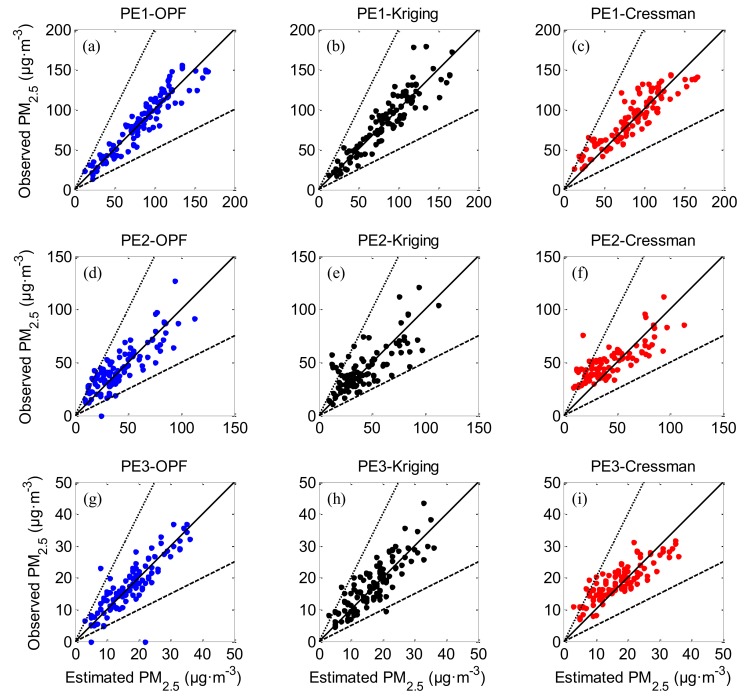
Comparison of estimated and observed PM_2.5_ concentrations in practical experiments (PE1: (**a**) to (**c**), PE2: (**d**) to (**f**), PE3: (**g**) to (**i**). OPF method: blue dots, kriging interpolation: black dots and Cressman interpolation: red dots. The 1:2, 1:1 and 2:1 lines are shown for reference.).

Figure 13 shows the comparison of estimated and observed PM_2.5_ concentrations for PE1, PE2 and PE3. The dotted, solid and dashed lines indicate the ratio of observed concentrations to estimated values equal to 2, 1, and ½, respectively. The results of the OPF method suggest that the estimated concentrations of PM_2.5_ agree well with the observations, with values concentrating near the solid line (the line indicating observations equal to estimations). Meanwhile, the interpolated results of kriging and Cressman methods are dispersedly distributed around that solid line, which involves relatively large deviations between the observed and estimated values.

**Figure 14:**
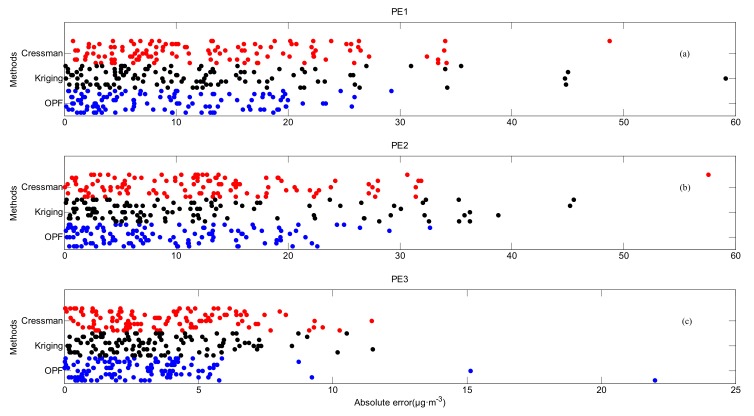
Absolute errors of cities in data set A for (**a**) PE1, (**b**) PE2 and (**c**) PE3 (OPF method: blue dots, kriging interpolation: black dots and Cressman interpolation: red dots).

Figure 14 demonstrates the absolute errors of cities in data set A for practical experiments. In PE1, errors from most of the cities are less than 20 μg·m^−3^ with the OPF method, whereas errors of kriging and Cressman interpolations are relatively larger, exceeding 30 μg·m^−3^ in a small number of cities. Similar to PE1, cities with errors larger than 30 μg·m^−3^ appear when kriging or Cressman interpolation is applied in PE2. In PE3, the large majority of errors with the OPF method are within 5 μg·m^−3^. Estimation by the OPF method is more accurate for the number of cities with errors less than 5 μg·m^−3^, which are apparently more than those with kriging or Cressman interpolation. 

**Figure 15:**
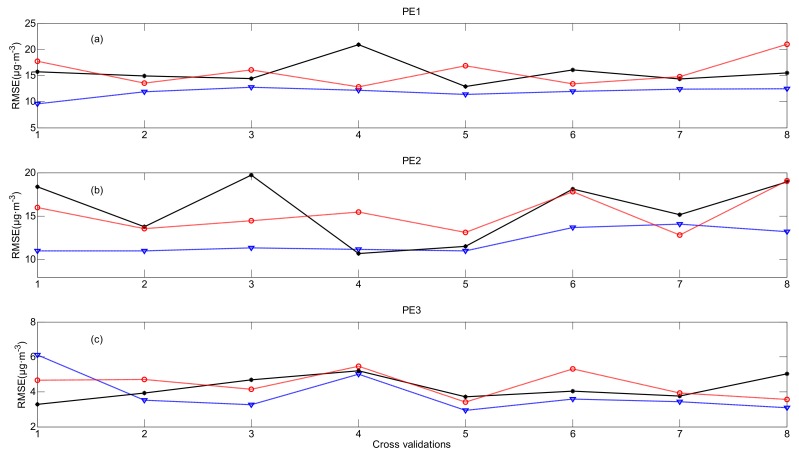
RMSEs in (**a**) PE1, (**b**) PE2 and (**c**) PE3 (OPF method: blue dashed line, kriging interpolation: black solid line and Cressman interpolation: red dash-dot line).

The RMSEs of practical experiments are shown in Figure 15. In PE1, the RMSEs of the OPF method are smaller than those of kriging and Cressman interpolations in all the cross-validation processes. In PE2, the results calculated by the OPF method are also more accurate in most of the cross-validation processes except for CV4 and CV7. The variation of RMSEs in PE3 is consistent with those in PE1 and PE2, indicating that the distribution of PM_2.5_ calculated by the OPF method is closer to the observation than those by calculated by kriging or Cressman interpolation.

## 4. Conclusions

In this paper, an orthogonal polynomial fitting method using Chebyshev basis functions is presented to estimate distributions of PM_2.5_ concentrations in the central and southern regions of China. Applying the OPF method requires three major steps. First, polynomial coefficients are computed based on monitoring data. Secondly, appropriate polynomial orders need to be selected. Finally, spatial distributions of PM_2.5_ fields are calculated. In the idealized twin experiments, IE1 is designed in accordance with the most common distribution of PM_2.5_ concentrations in China, whereas IE2 represents a common air contamination pattern in spring and autumn. Distributions of PE1 and PE2 are successfully recovered by the OPF method with accuracy higher than kriging and Cressman interpolations. Therefore, it is validated that the OPF method is reliable in estimating the spatial distribution of PM_2.5_ concentrations. In the practical experiments, PE1 suggests that errors are the smallest when fitting orders of *x* and *y* directions are both set to 6 for the OPF method. The amount of data is insufficient to support high-order OPF. The results of the practical experiments also indicate that the OPF method can reconstruct the PM_2.5_ fields accurately. The estimated PM_2.5_ concentrations computed by the OPF method are closer to the observations. The spatially averaged MAEs of the OPF method are lower than those of kriging or Cressman interpolations in all the practical experiments. The RMSEs of the OPF method in the practical experiments are the smallest in most of the cross-validation processes investigated among those methods.

## Appendix A. OPF Method

In our study, the OPF method is based on Chebyshev basis functions. Let $x_{i}$ be the points on the *x* axis (i=1,2,⋯,n). The Chebyshev polynomials are:

(A1) $$\begin{array}{l} \Phi_{0}{(x}_{i})=1,\\ \Phi_{1}{(x}_{i}{)=x}_{i}{-P}_{1,0}\Phi_{0}{(x}_{i}),\\ \Phi_{2}{(x}_{i}{)=x}_{i}{}^{2}{-P}_{2,1}\Phi_{1}{(x}_{i}{)-P}_{2,0}\Phi_{0}{(x}_{i}),\\ \ldots \\ \Phi_{k}{(x}_{i}{)=x}_{i}{}^{k}{-P}_{k,k-1}\Phi_{k-1}{(x}_{i}{)-P}_{k,k-2}\Phi_{k-2}{(x}_{i})-\ldots {-P}_{k,0}\Phi_{0}{(x}_{i}), \end{array}$$

where *k* is the order of polynomials in the *x* direction, $\phi_{0}\left(x_{i}\right),\;\phi_{1}\left(x_{i}\right),\ldots ,\;\phi_{k}\left(x_{i}\right)$ are Chebyshev polynomials with orders from zero to *k* and *P* is the coefficient of Chebyshev polynomials, calculated as:

(A2) $$P_{k,l}=\frac{\sum_{i=1}^{n}x_{i}^{k}\Phi_{l}{(x}_{i})}{\sum_{i=1}^{n}\Phi_{l}^{2}{(x}_{i})},$$

where $P_{k,l}$ is the *l*-th coefficient of the *k*-order polynomial. The distribution of PM_2.5_ concentrations $Z\left(x_{i},y_{j}\right)$ can be fitted as:

(A3) $${\tilde{Z}(x}_{i}{,y}_{i})=\sum_{k=0}^{K_{0}}\sum_{s=0}^{S_{0}}A_{k,s}\Phi_{k}{(x}_{i}{)\varsigma }_{s}{(y}_{j}),$$

where *i* = 1,2,…,*N*, *j* = 1,2,…,*M*, k and s are the orders of polynomials for the *x* and *y* directions respectively, $K_{0}$ and $S_{0}$ are the corresponding cutoff orders. $A_{k,s}$ are expansion coefficients. $\phi_{k}\left(x_{i}\right)$ is the *k*-order Chebyshev orthogonal polynomial in the *x* direction, and $\varsigma_{s}{(y}_{j})$ is the *s*-order Chebyshev orthogonal polynomial in the *y* direction. $\tilde{Z}\left(x_{i},y_{j}\right)$ is the fitted value for function Z. The sum of squares of the error ε is:

(A4) $${\varepsilon (A}_{0,0}{,A}_{1,0},\ldots {,A}_{k_{0}s_{0}})=\sum_{i=1}^{N}\sum_{j=1}^{M}[Z-\sum_{k=0}^{K_{0}}\sum_{s=0}^{S_{0}}A_{k,s}\Phi_{k}{(x}_{i}{)\varsigma }_{s}{(y}_{j}{)]}^{2}.$$

According to the Rolle intermediate value theorem, we obtain:

(A5) $$\frac{\delta \varepsilon }{\delta A_{m,n}}=2\sum_{i=1}^{N}\sum_{j=1}^{M}{[Z}_{i}-\sum_{k=0}^{K_{0}}\sum_{s=0}^{S_{0}}A_{k,s}\Phi_{k}{(x}_{i}{)\varsigma }_{s}{(y}_{j}{)]\Phi }_{m}{(x}_{i}{)\varsigma }_{n}{(y}_{j})=0.$$

Owing to the orthogonality of Chebyshev polynomials:

(A6) $$\begin{array}{l} \sum_{i=1}^{N}\Phi_{k}{(x}_{i}{)\Phi }_{m}{(x}_{i})=0,\;k\neq m,\\ \sum_{j=1}^{M}\varsigma_{s}{(y}_{j}{)\varsigma }_{n}{(y}_{j})=0,\;s\neq n, \end{array}$$

where *m* and *n* are the orders of polynomials for the *x* and *y* directions, respectively. Combining Equation (A5) with Equation (A6), the expansion coefficients can be solved as:

(A7) $$A_{k,s}=\frac{\sum_{i=1}^{N}\sum_{j=1}^{M}{Z(x}_{i}{,y}_{j}{)\Phi }_{k}{(x}_{i}{)\varsigma }_{s}{(y}_{j})}{\sum_{i=1}^{N}\Phi_{k}^{2}{(x}_{i})\sum_{j=1}^{M}\varsigma_{s}^{2}{(y}_{j})}.$$
